# How Human Papillomavirus Replication and Immune Evasion Strategies Take Advantage of the Host DNA Damage Repair Machinery

**DOI:** 10.3390/v9120390

**Published:** 2017-12-19

**Authors:** Valentina Bordignon, Enea Gino Di Domenico, Elisabetta Trento, Giovanna D’Agosto, Ilaria Cavallo, Martina Pontone, Fulvia Pimpinelli, Luciano Mariani, Fabrizio Ensoli

**Affiliations:** 1 Clinical Pathology and Microbiology Unit, San Gallicano Dermatology Institute, IRCCS, IFO, Via Elio Chianesi 53, 00144 Rome, Italy; enea.didomenico@ifo.gov.it (E.G.D.D.); elisabetta.trento@ifo.gov.it (E.T.); giovanna.dagosto@ifo.gov.it (G.D.); ilaria.cavallo90@gmail.com (I.C.); martina.pontone@ifo.gov.it (M.P.); fulvia.pimpinelli@ifo.gov.it (F.P.); fabrizio.ensoli@ifo.gov.it (F.E.); 2 HPV Unit, Department of Gynaecologic Oncology, National Cancer Institute Regina Elena, IRCCS, IFO, Via Elio Chianesi 53, 00144 Rome, Italy; luciano.mariani@ifo.gov.it

**Keywords:** human papillomavirus (HPV), DNA damage repair (DDR), ATM, ATR, STAT-5, IFN-γ, viral immune evasion

## Abstract

The DNA damage response (DDR) is a complex signalling network activated when DNA is altered by intrinsic or extrinsic agents. DDR plays important roles in genome stability and cell cycle regulation, as well as in tumour transformation. Viruses have evolved successful life cycle strategies in order to ensure a chronic persistence in the host, virtually avoiding systemic sequelae and death. This process promotes the periodic shedding of large amounts of infectious particles to maintain a virus reservoir in individual hosts, while allowing virus spreading within the community. To achieve such a successful lifestyle, the human papilloma virus (HPV) needs to escape the host defence systems. The key to understanding how this is achieved is in the virus replication process that provides by itself an evasion mechanism by inhibiting and delaying the host immune response against the viral infection. Numerous studies have demonstrated that HPV exploits both the ataxia-telangiectasia mutated (ATM) and ataxia-telangiectasia and rad3-related (ATR) DDR pathways to replicate its genome and maintain a persistent infection by downregulating the innate and cell-mediated immunity. This review outlines how HPV interacts with the ATM- and ATR-dependent DDR machinery during the viral life cycle to create an environment favourable to viral replication, and how the interaction with the signal transducers and activators of transcription (STAT) protein family and the deregulation of the Janus kinase (JAK)–STAT pathways may impact the expression of interferon-inducible genes and the innate immune responses.

## 1. Introduction

Mammals have evolved sophisticate innate and adaptive immune mechanisms to control local and systemic viral infections and to limit damage to the host when infection persists [1].

Once a virus establishes an infection, the host immediately turns on shelter mechanisms aimed at neutralizing and, possibly, eliminating the pathogen. In turn, viruses have evolved survival mechanisms aimed at subverting these defences.

The human papilloma virus (HPV) is especially capable of preventing a robust immune response and thus is very slow to clear [2]. The most important strategies to evade host surveillance are aimed at suppressing important inflammatory immune pathways or at becoming invisible to the host immune surveillance. These strategies include a limited, almost absent triggering of danger signals, as HPV infection causes no cytolysis, no cytopathic cell death, and no inflammation. In addition, the suppression of the interferon (IFN) response, the resistance to immune-mediated apoptosis, and the downregulation of adhesion molecules for antigen-presenting cells (APCs) dampen a whole network of genes downstream of the pattern recognition receptors (PRRs, such as Toll-like receptors (TLRs) and retinoic acid-inducible gene I (RIG-I)-like receptors), while the impaired production of active major histocompatibility complex (MHC) class I and II components of the antigen-presenting machinery [3,4] contributes to minimizing the exposure of viral antigens to immune and epithelial cells. 

In order to successfully replicate, HPV exploits the DNA Damage Repair (DDR) machinery of the host cells. The DDR machinery includes a vast network of proteins evolved to protect genomic integrity in response to endogenous and exogenous insults. Many viruses were shown to make use of the DNA repair pathways in order to efficiently replicate [5,6]. HPV is able to recruit DNA repair factors necessary for viral genome replication [7], specifically through the activation of the ataxia-telangiectasia mutated (ATM) and the ataxia-telangiectasia and rad3-related (ATR) DNA damage kinases [8]. Different DDR pathways protect the host cell genome integrity by targeting HPV episomal DNA for elimination. For instance, DNA-binding, N-methylpyrrole-imidazole hairpin poly-amides (PAs) were shown to target HPV DNA and destabilize and reduce, or eliminate HPV episome levels in infected cells. This evidence should be further explored also considering its potential for application for novel antiviral strategies against HPV [9,10]. 

## 2. The DDR Machinery

Depending on the type of damage mechanism, cells can rely on a variety of repair pathways. The three major regulators of the DDR are ATM, ATR, and DNA-dependent protein kinase (DNA-PK) kinases, which belong to the phosphatidylinositide 3-Kinase (PI3KK) family of kinases [11].

DNA damage often results in DNA mutations, crosslinking, single-strand breaks (SSBs), as well as double-strand breaks (DSBs) [12]. To repair these damages, cells can activate various pathways, including base excision repair (BER), nucleotide excision repair (NER), mismatch repair, non-homologous end joining (NHEJ), homologous recombination repair (HRR), and interstrand crosslink repair [13]. Among these, NHEJ and HRR are the two major repair mechanisms for DSBs in eukaryotes, while BER enzymes are major players for SSB repair [14]. NER is an important component of the DDR capable of maintaining genome integrity against genotoxic insults through the removal of a wide spectrum of DNA helix-distorting lesions. The NER machinery excises bulky DNA lesions from dsDNA resulting in a single-stranded gap that is filled in and ligated. Following DNA insults, various complexes of cellular proteins are recruited to DNA damage loci to activate DNA damage-responsive PI3K-like Ser/Thr kinases, which consist of ATM, ATR, and DNA-PK.

The importance of DDR proteins is not limited to protecting the genomic material, since many DDR proteins also play key roles in supporting an effective immune response. 

A subset of DDR proteins participates in the recombination of genes coding for antibodies, thus supporting the generation of the almost unlimited diversity of antibody responses [15]. Additionally, many of these proteins play a part both in the cellular response to viral infections as well as in the life cycle of multiple viruses. In some cases, such as adenovirus infections, host DDR pathways act to restrict viral propagation [16,17], and, predictably, these viruses that are adversely affected by the host DDR machinery have evolved mechanisms to subvert it [6,18,19]. On the opposite, other viruses, such as members of the herpes virus, polyoma virus, and papilloma virus families, rely on the host DDR response to replicate their genomes [20,21,22,23]. This latter strategy involves the activation of DDR proteins and their recruitment to virus replication centers, thus providing access to DDR-associated polymerases that are independent of origin licensing requirements [24,25,26,27,28,29].

## 3. HPV Life Cycle

**Figure 1:**
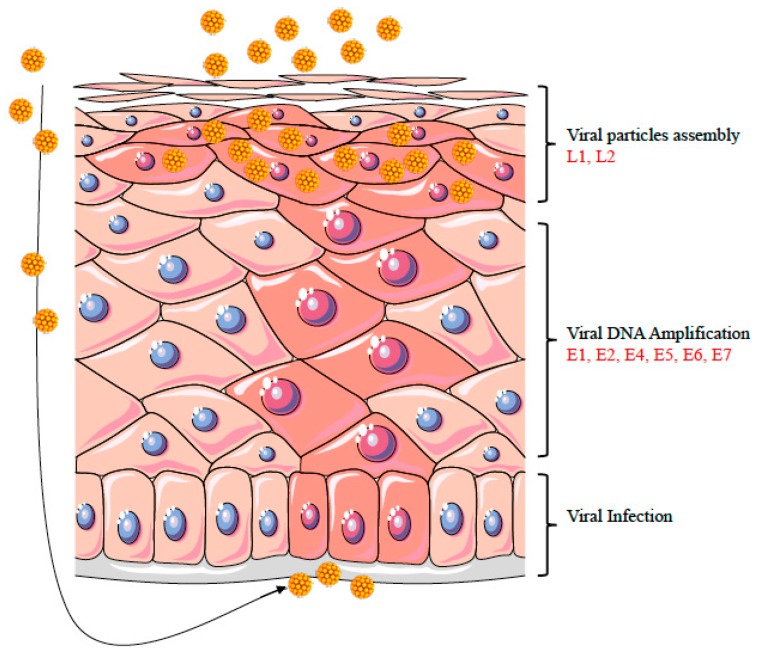
Epithelial differentiation triggers the productive phase of the HPV life cycle. The figure shows a diagram of a stratified epithelium. HPV-infected cells are shown with pink nuclei. HPV infects the basal keratinocytes of the stratified epithelium through a microwound (as shown by the black arrow on the left). Epithelial differentiation triggers the amplification phase of HPV life cycle, followed by late gene expression and virion assembly. The increased HPV replication is shown in the diagram by more numerous and larger pink circles, which indicate an increase of both viral replication centers and DNA damage foci.

HPVs are double-stranded DNA viruses that preferentially infect mucosal or cutaneous stratified squamous epithelia. Mucosal-tropic HPV subtypes are classified as high-risk or low-risk, according to their association with cancer. Clinical manifestations of infection by low-risk HPV subtypes may progress from completely asymptomatic to the development of different types of benign papillomas or warts. Conversely, high-risk HPV types are recognized as etiological agents of cervical and anogenital malignancies as well as of different form of oropharyngeal cancers [30]. During infection, HPVs escape immune surveillance and can remain latent for decades. Although there is a mounting evidence that the DDR is important for HPV genome amplification, less is known about how HPV regulates it to accomplish its life cycle. The HPV life cycle has evolved to contend with the distinct cell states found in a differentiating epithelium and relies on cellular factors [31]. The life cycle of HPVs depends on epithelial differentiation (Figure 1). Since HPVs do not encode their own DNA polymerases and other accessory factors, their replication is largely dependent on the host machinery, including transcriptional factors, microRNAs (miRNAs), kinases, apoptotic caspases, epigenetic enzymes, and DNA damage signalling [32]. Evidence suggests that HPV activates an ATM-dependent DDR to amplify viral genomes in a recombination-dependent manner, which, in turn, is supported through the recruitment of DDR repair proteins to viral replication compartments [33,34,35]. Such diversion of the host biochemical machinery is achieved by the expression of a complex set of viral proteins. Namely, the early gene products E1 and E2 activate the DDR and recruit repair proteins to viral replication centers, where they are likely needed to replicate the viral genome. Viral proteins E6 and E7 represent two important oncogenes, which act on the cell cycle by targeting hinge proteins of the cell cycle control point (i.e., p53 and retinoblastoma protein (pRb), respectively). Further, E6 and E7 prevent the DDR response from pausing cell cycle progression or apoptosis and are necessary for both HPV genome maintenance and amplification. Viral gene products E4 and E5 act by regulating late viral functions, playing a role in virion release and immune evasion, respectively, and are also required for productive viral replication. Late gene product L1 and L2 are structural proteins which concur in assembling the mature viral capsid.

HPV infection primarily involve the basal cells of the layered epithelium, where the virus establishes a reservoir mainly characterized by episomal viral DNA forms and low levels of gene expression. Epithelial differentiation triggers the replicative HPV cycle, leading to the productive phase with the generation of thousands of viral copies per infected cell, the subsequent expression of late genes, and the virion assembly. The amplification of the viral genome follows the synthesis of cellular DNA during the cell transition from phase S to a G2-like phase [36], thus providing the cellular factors necessary for viral replication. While replication maintenance occurs through a bidirectional theta mode, increasing evidence suggests that the viral productive replication takes place through a distinct manner from what is found in undifferentiated cells [37].

## 4. Normal Immune Response in the Uterine Cervix

HPVs induce chronic infections that have virtually no systemic clinical manifestations, rarely kill the host, and periodically shed large amounts of infectious virus for transmission to naive individuals. Such an evolutionarily successful lifestyle is achieved by the virus replication strategies, which allow HPVs to evade the host defence systems.

In the cervical tissue, the cells of the innate immune system, namely macrophages, Langerhans’ cells (LCs), dendritic cells (DCs), neutrophils, natural killer (NK) cells, γδ T lymphocytes, and keratinocytes, recognize foreign structures by means of receptors such as Toll-like receptors (TLRs), whose signalling activate a cascade of inflammatory cytokines and chemokines, such as the interleukins (ILs) IL1β, IL6, IL8, IL12, tumor necrosis factor (TNFα), and α-, β-, and λ-Interferon (IFN). These events aim at the recruitment of immune cells and the secretion of antimicrobial factors, effectively linking innate and adaptive immunity [38]. Upon antigen recognition and inflammatory cytokines production, DCs, which are major resident APCs, process the antigen and upregulate the expression of the major histocompatibility complex (MHC; HLA in humans) for presenting the processed antigen to T cells. For this task, DCs migrate to T cell areas of secondary lymphoid tissues where they undergo a final maturation, which allows their binding to receptors expressed on antigen-specific T cells [39]. DC–T cells binding results in the induction of T cell proliferation, IL-2 production, and upregulation of anti-apoptotic genes, which represent the molecular basis of the adaptive immune response. It should be noted that antigen recognition in the absence of costimulatory molecules results in T cell anergy as well as in the binding of the inhibitory receptor cytotoxic T lymphocyte Antigen 4 (CTLA-4) to CD80 [40]. The responses of activated CD4^+^ T cell (including the pattern of cytokine production) may follow different pathways, including a cell-mediated Th1 response (characterized by the production of IL2, IL12, IL15, TNF-α, and γ-IFN), a humoral Th2 response (characterized by IL4, IL5, IL6, IL10, and IL13), a Treg/Th3 regulatory response (characterized by IL10, TGF-β, γ-IFN), and a Th17 response (characterized by IL17, IL23, and IL32). On the other hand, the activation of CD8^+^ T cells generates cytotoxic T lymphocytes (CTL) capable of recognizing and killing infected cells through the secretion of proteolytic enzymes (i.e., granzyme and perforin) [41].

## 5. HPV and DDR: Strategies to Escape Immune Surveillance

**Figure 2:**
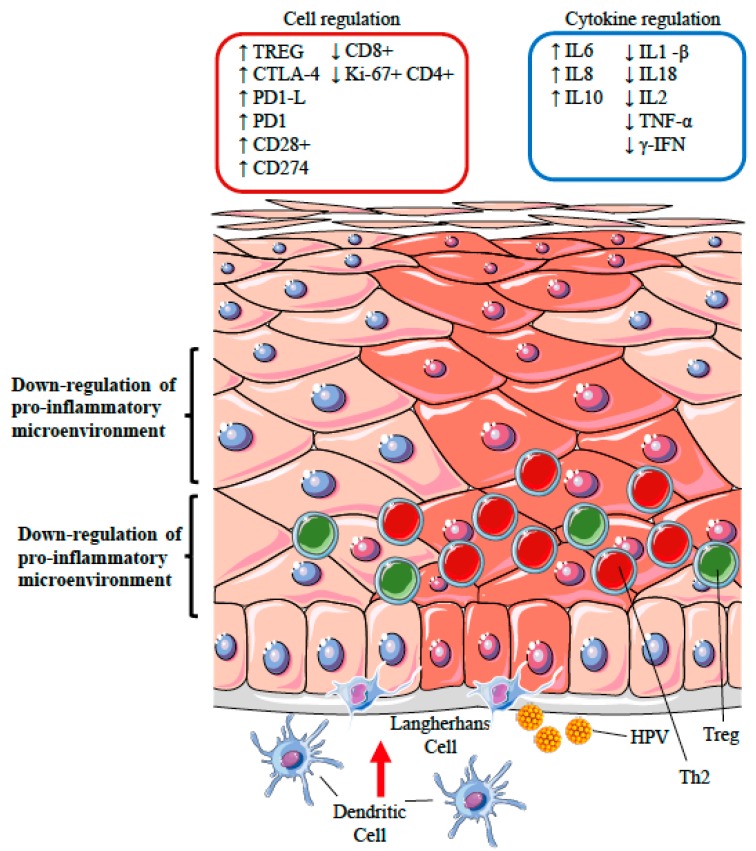
Schematic representation of the HPV strategies likely to modify the immunological microenvironment in the site of infection. HPV has evolved mechanisms both to avoid initial recognition by the immune system as well as to interfere with adaptive immunity. A primary mechanism of viral immune evasion is likely achieved by avoiding antigen processing and presentation by Langerhans and dendritic cells (represented in the diagram as a lack of migration of these cells from the dermis). Further, HPV has evolved mechanisms capable of inhibiting key host antivirus natural and adaptive responses, including the modulation of the cascade of inflammatory or immunoregulatory cytokines and chemokines, IFN production, as well as the activity of cytotoxic T cells and natural killer cells, and the humoral antibody response. The recruitment of CD4^+^ CD25^+^ regulatory T cells (Tregs, depicted as green circles) and the presence of activated Th2 cells (depicted as red circles), can lead to a further suppression of cytotoxic functions, induction of T cell anergy, and apoptosis. The pink nuclei identify the HPV-infected epithelium. The up and down arrows represent the increased or decreased expression of cell markers or cytokines.

HPV has evolved mechanisms both to avoid initial recognition and to interfere with adaptive immunity. In fact, HPV needs to persist in squamous epithelia for a certain amount of time to complete its reproductive life cycle. This event represents the most important risk factor for the development of HPV-associated premalignant lesions and may lead to HPV-driven cancers [3,42]. The primary mechanism of viral immune evasion for HPV infection is likely represented by the avoidance of antigen processing and presentation, the shifting from a Th1 to a Th2 immune response, the silencing of the inflammatory response through the recruitment of regulatory T cells, and the modulation of apoptosis [43] (Figure 2). However, one of the most important mechanisms demonstrating the hijacking of the DDR and immune subversion consists of the dysregulation of IFN responses [44].

## 6. Deregulation of Interferon Synthesis

Like most DNA viruses, HPVs have also developed effective strategies to prevent IFN synthesis by the deregulation of the STAT family that contains important regulators of the innate immune response [20,34]. Upon viral infection, E6 and E7 directly influence the expression of IFNα and IFNγ because they are able to inhibit interferon-regulatory factors 1 and 9 (IRF1, IRF9), and directly interfere with the JAK–STAT pathway. In addition to the interferon pathway, HPV can also dampen multiple genes downstream of the receptor recognition pathway (i.e., PRR, such as TLR and RIG). These include antimicrobial molecules, chemiotactic and proinflammatory cytokines, as well as the inflammasome and molecules involved in antigen processing [45]. 

## 7. STAT Signalling is Part of the IFN Pathway

Type I interferons, namely IFN-α and IFN-β, act as a bridge between innate and adaptive immunity, thanks to their antiviral, antiproliferative, antiangiogenic, and immunostimulatory properties [46].

By interacting with components of the interferon signalling pathways, high-risk HPV types downregulate IFN-α-inducible gene expression. In particular, E7 oncoprotein inhibits IFN-α-mediated signal transduction by binding to P48/IRF-9, thus preventing its translocation to the nucleus and thereby inhibiting the formation of the ISGF-3 transcription complex that binds the interferon-specific response element (ISRE) in the nucleus [47]. When physically associated with IRF-1, E7 is able to inhibit IRF-1-mediated activation of the IFN-β promoter and, thereby, the recruitment of a histone deacetylase to the promoter, thus inhibiting its transcription [48]. 

This notion is further supported by in vivo expression of HPV18 E7. In fact, E7 is able to inhibit the transactivating function of IRF-1 by reducing the expression of IRF-1 target genes, such as *TAP1*, *IFN-β* and *MCP-1* genes [49]. Similarly, the interferon pathway is also hit by the E6 protein of HPV. E6 binds the transcriptional activator IRF-3 and thereby prevents the transcription of IFN-α mRNA [50]. Moreover, E6 specifically inhibits IFN-α-mediated signalling by impairing JAK–STAT activation and the phosphorylation of tyrosine kinase 2 (TYK2), STAT1, and STAT2 [51].

## 8. STAT-5 Activation Promotes Both ATM and ATR Signalling

One of the primary pathways regulating the innate immune response is JAK–STAT [52]. Epigenetic signals, including growth factors and cytokines such as IFNs, induce the translocation of STAT proteins to the nucleus, which triggers the activation of the JAK–STAT pathway. This leads to the increased expression of multiple downstream genes [53,54]. STAT-1, -2, -3, -4, -5, and -6 are different proteins belonging to the STAT family. Recent evidence has shown that the suppression of STAT-1, but not STAT-2 or STAT-3 transcription by HPV gene products, is necessary for the stable maintenance of viral episomes and genome amplification [55], while STAT-5 is necessary for HPV genome amplification in differentiating cells through the induction of the ATM DNA damage pathway [34]. 

STAT-5 regulates the expression of a set of downstream genes. The mechanism by which this transcription factor activates or suppresses the ATM kinase pathway may involve either a direct interaction with a kinase such as JAK2 or, alternatively, an interaction with some upstream regulator of ATM activity, through the deregulation of the acetyltransferase TIP60 [56]. Another factor that is regulated by STAT-5 is the peroxisome receptor, namely, the peroxisome proliferator-activated receptor gamma (PPARγ), that acts as an intermediary in activating the ATM DNA damage response through the regulation of p63 expression [57] and has been suggested to be a regulator of the DNA damage response [58].

Evidence indicates that the JAK–STAT transcriptional regulator STAT-5, that is necessary to induce the DNA damage response, is involved in HPV genome amplification. This suggests that HPV proteins differentially activate and suppress members of the JAK–STAT pathway to allow for a differentiation-dependent productive replication by the modulation of the ATM DNA damage pathway.

## 9. HPV and Genomic Instability: Modulation of Apoptosis

Apoptosis is a mechanism of programmed cell death by which the host eliminate damaged or infected cells [31]. HPV has evolved several mechanisms to deregulate host cell cycle and to control apoptosis, facilitating the accumulation of DNA damage and, consequently, cellular transformation. Different HPV proteins can activate the cellular DDR response. DDR activation leads the cell to pause the cell cycle in order to facilitate DNA damage repair. However, the HPV E7 protein inhibits cell cycle arrest, thus preventing DNA repair. On the other hand, by decoupling DDR signalling from apoptotic signalling, the E5 and E6 proteins inhibit cellular apoptosis, which is normally triggered by the accumulation of extensive DNA damage, therefore increasing the accumulation of DNA alterations. 

In the high-risk HPV strains E5, E6, and E7 are key oncoproteins with antiapoptotic properties, which are considered the main contributors to malignant transformation [59].

E5 protein is the smallest HPV oncoprotein, spanning about 90 amino acids in length [60], that can localize into different intracellular membranes, including the Golgi apparatus and the endoplasmic reticulum (ER), or in the nuclear membrane. 

In HPV16 and 18 infection, E5 mRNA and protein were detected in anogenital low-grade squamous intraepithelial lesions, protecting infected cells from apoptosis in the early steps of infection. The mechanism by which HPV E5 protects cells from apoptosis relies on two main pathways, i.e., the inhibition of both death receptor-mediated apoptosis and ER stress-induced apoptosis [61]. Specifically, in HPV 16, E5 down regulates tumour necrosis factor ligand (FasL) and tumor necrosis factor-related apoptosis-inducing ligand (TRAIL)-mediated apoptosis by decreasing the total amount of Fas receptor and reducing Fas surface location. This prevents the formation of the Death-Inducing Signalling Complex (DISC), impairing the activation of the downstream effectors caspases 3 and 7 and consequently inhibiting apoptosis mediated by TRAIL [62]. The presence of exogenous viral proteins may also activate cellular defence mechanisms inducing ER stress [63]. Therefore, E5 downregulates the three main proteins in the ER stress pathway: cyclooxygenase-2 (COX-2), X-box binding protein 1 (XBP-1), and inositol-requiring enzyme-1α (IRE1α) [64]. The reduced activity of COX-2, XBP-1, and IRE1α, promotes viral replication and persistence [65], contributing to the development of cancer in high-risk HPVs [66]. In addition to the relevant role of E5, in vivo and in vitro studies have identified E6 and E7 as the main HPV oncogenes. 

## 10. HPV E6 and p53

The best characterized mechanism by which HPV E6 inhibits apoptosis is by degrading or otherwise inactivating p53 [67]. Consequently, the downregulation of p53 indirectly promotes chromosomal instability, thus increasing the probability of the infected cells to progress towards malignancy [68]. 

The protein p53 is a DNA site-specific transcription factor that is activated in response to stress or DNA damage and positively regulates the expression of genes involved in the control of cell cycle arrest or apoptosis [69]. The HPV E6 is an oncoprotein of 150 amino acids that has no enzymatic activity but has the potential to trigger protein–protein binding [70]. Indeed, E6 interacts with E6-associated protein (E6AP), which is a ubiquitin ligase. The E6–E6AP complex binds p53, which becomes very rapidly ubiquitinated and targeted to proteasomes for degradation [71]. 

Underlining the necessity for HPV to avoid the activation of p53, HPV E6 not only promotes p53 degradation, but also independently inhibits any remaining p53 from transactivating its target genes in response to DNA damage [72].

Furthermore, HPV E6 also blocks p53 transactivation indirectly by inhibiting proteins that activate p53 in trans. In response to DNA damage, p53 transactivation activity is enhanced by the acetylation of p53 that increases p53 DNA binding affinity. HPVE6 binds three regions of the closely related histone acetyltransferases (HATs) and inhibits p53 acetylation and transactivation [73,74].

E6 is also able to block apoptosis in cells and mice lacking p53 by regulating apoptosis through a p53-independent mechanism that protects the infected cells from multiple apoptotic stimuli [75]. Another major function of E6 is the ability to activate the expression of the catalytic subunit of the human telomerase reverse transcriptase (hTERT), which is critical for cell immortalization. In fact, hTERT expression and telomerase activity are higher in cells expressing both E6 and E7 than in cells expressing E6 alone. This indicates that E7 augments E6-mediated activation of hTERT transcription [76].

## 11. HPV E7 and pRB

Similarly to E6, the E7 oncoprotein, a polypeptide composed of approximately 100 amino acids, inhibits p53-dependent and -independent apoptosis [77]. The E7 protein is considered the major HPV oncoprotein and its expression is necessary for viral pathogenesis and cellular transformation. The main host targets of E7 are pRb and its associated proteins, p107 and p130. pRb is a central regulator of multiple cellular processes including cell cycle, differentiation, and apoptosis. The HPV E7 oncoprotein targets pRb for proteasomal degradation overcoming its growth inhibitory function [78]. E7 induces the degradation of pRb, resulting in the activation of several transcriptional regulators, such as E2F, Elf-1, and the cyclin-dependent kinases (CDK), which promote cell cycle progression, rendering the cell independent of any type of control [79]. 

In addition to the well-characterized target pRb, the HPV E7 oncoprotein also affects ATM, a key regulator of cell cycle progression, telomere maintenance, and apoptosis [80]. In differentiating cells, the ATM-dependent DNA damage response mediated by E7 induces an S or G2/M arrest that is necessary for the efficient replication of the viral genomes [81]. 

## 12. HPV and the Immune System: Subversion and Piracy

### 12.1. Perturbation of Antigen Processing and Presentation

The key viral strategy to avoid the host immune response is to mask itself from immune surveillance. Indeed, HPV infects exclusively epithelial cells, and the whole virus replicative cycle resides outside the basement membrane, therefore away from the resident dermal immune effector cells. The expression of viral proteins appears only to occur in the keratinocytes of the upper layers of the epithelium, where the immune system has a limited access. Furthermore, virus shedding from the infected cells does not induce any detectable viremia [1] and is not accompanied by cell death or cell lysis. In fact, the expression of the virus capsid proteins (L1–L2) is limited to the superficial epithelial cells. This mechanism, which is partly based on the preferential HPV usage of codons that mammalian cells rarely use, leads to a diminished production of capsid proteins in the basal cells, because of the unavailability of the corresponding tRNA [82] and may therefore reduce the recognition of capsid proteins by the immune system. As noted, in contrast to most viruses, HPV does not kill epithelial cells and, as a result, APCs cannot engulf virions and effectively present the antigens to the immune cells. In addition, the E7 and E6 non structural proteins of the high-risk HPV16 virus subtype have pleiotropic activities, including the capability to delay cell differentiation and impair immune functions [1]. Furthermore, HPV16 E7 is expressed in keratinocyte nuclei where it is inaccessible to APCs and uses motifs common to human proteins, which further helps to evade antigen recognition [83]. These mechanisms are also thought to lead to an impaired migration of Langerhans cells to the skin [84]. HPV interference with antigen processing and presentation includes also a decreased expression of the proteasome subunits low-molecular mass polypeptide 2 (LMP2) and LMP7, a decreased expression of transporter subunits (TAP1 and TAP2), and a reduced expression of MHC-I itself. In fact, the E7 protein was found to repress the MHC class I heavy chain gene promoter. The mechanism relies on the arrest of MHC class I molecules in the Golgi apparatus and in the breakdown of the chaperone involved in the maturation of MHC class II, thus leading to the inhibition of its expression on the cellular surface. The functional effect of the downregulation of MHC class I and class II molecules on the surface of HPV-infected cells consists of a reduced recognition by T cells, which, in turn, leads to an escape of HPV from immune surveillance. Moreover, the E5 protein has been shown to enhance the expression of gangliosides on cervical epithelial cells [85], which might be expected to inhibit cytotoxic T cell function locally. Additionally, HPV E6 and E7 have been implicated in the direct inhibition of TLR9-mediated pathways by downregulating the transcription of the *TLR9* gene, a key proinflammatory signalling molecule [86]. 

### 12.2. Polarization of T Cell Phenotypes

A further immune escape mechanism employed by HPV is the inhibition of the Th1-inflammatory and cytotoxic response by the induction of a shift from a Th1 cellular response to a Th2 humoral response. 

HPV-related lesions were found to be characterized by weak or absent IFNγ-associated Th1 cell responses and by an upregulation of Th2 cytokines (including IL-6, IL-8, and IL-10) [87]. In fact, cervical cancer progression was associated with a Th1 to Th2 shift of cytokine expression, which appears to be induced by two E7-derived epitopes and by an increased IL10 expression [88]. CD4^+^ T cells from HPV-associated lesions showed impaired production of IL-1β, IL-18, IL2, IFN-γ, TNF-α, and the frequency of Ki-67^+^ CD4^+^ T cells was significantly reduced as compared to primary papillomas. Moreover, CD1d expression was changed in HPV-infected cells. CD1d is an important player in innate immune responses and can modulate the adaptive immune cells by altering the Th1/Th2 polarization [89]. HPV was shown to induce the expression of cytotoxic T lymphocyte Antigen 4 (CTLA-4) and of programmed cell death 1 (PD-1, CD28) on T and B activated cells. At the same time, E proteins are capable to induce the production of PD-1 Ligand (PD-1L, CD274) on antigen-presenting cells and on tumor cells. By these means, HPV can effectively suppress cytotoxic functions and immune surveillance. The interaction between PD-1 and its ligand (PD-1–PD-1L pathway) induces T cell anergy and apoptosis. Notably, the frequency of PD-1^+^ T cells and PD-1L on tissue lesions is a prognostic factor for malignant tumors, and the frequency of PD-1^+^CD4^+^ T cells is significantly increased in HPV lesions [90].

### 12.3. Silencing of the Inflammatory Response: the Role of Regulatory T Cells

Particular interest has been focused on regulatory T cells, identified by a CD4^+^ CD25+ FoxP3^+^ phenotype, within HPV lesions [91], and on the production of immunoregulatory cytokines, which might impact cytotoxic T cell function [92]. CD4^+^ CD25^+^ regulatory T cells (Tregs) play key roles in the homeostasis of the immune system, including immune tolerance as well as the response to infections and tumours. Tregs are able to control the production of inflammatory cytokines and prevent the proliferation and activation of naive CD4^+^ T cells, natural killer cells (NK), and Cytotoxic T lymphocytes (CTLs). By IL-10- as well as TGFβ-dependent mechanisms, Tregs can exert specific suppressive functions on the immune response. An increased number of Tregs with a strong immunosuppressive activity was found both at the tumour site and in the draining lymph node stations in cervical cancer patients. This evidence suggests that Treg lymphocytes may likely contribute to the impairment of the HPV-specific immune responses found in these patients. Indeed, high Treg frequencies appear to be strongly associated with an increased risk of progression from premalignant lesions to cancer, since a low ratio CD8^+^/Tregs is associated with a poor prognosis [93]. However, it is still unclear whether a persistent HPV infection is responsible for increased numbers of Tregs, or whether an increased Treg frequency leads to persistence. Moreover, Tregs specific for E6 and E7 antigens were identified in colorectal carcinoma (CC) and high-grade squamous intraepithelial lesions (HSILs) infiltrating lymphocytes, whereas in animal models it was shown that Treg depletion leads to antitumor immune responses [94].

## 13. Methods

The present review focuses on describing molecular mechanisms and circuitries involved in the process of HPV replication and how their deregulation can lead to immune evasion and carcinogenesis. 

The in-depth knowledge of the mechanisms for HPV-driven immune evasion is of utmost importance to help the design of novel, effective immunotherapies aimed at restoring an effective immune response. The reference list was updated in September 2017 and obtained from the PubMed database.

## 14. Conclusions

The multifaceted role of the DDR in viruses life cycle has recently become the focus of research. We discussed evidences regarding the complex role of the DNA damage response machinery in the HPV life cycle, which link the immune regulator STAT-5 and the ATM–ATR pathways in the mechanisms of HPV genome amplification and immune deregulation. Cancers are generally regarded as genetic diseases influenced by environmental risk factors. In the case of HPV infection, malignancies arise from a complex interaction between the virus and the host. Viral proteins induce host cells to drive an effective viral production, while evading the host immune defence. 

Improving our understanding of the strategies evolved by HPV to overcome the host defence mechanisms may provide important insights to advance the development of new therapeutic strategies against HPV-related diseases.
